# The Key-Note to Success

**Published:** 1895-11

**Authors:** 


THE KEY-NOTE TO SUCCESS.—The JOURNAL has time and again preached the doctrine that if the medical profession in Texas ever hope to secure the much needed medical legislation to rid the State of quackery, each member must consider himself a committee of one especially instructed to "work upon" his immediate representative, or the candidate for representative from his county; to make a convert of him to the cause; to make him see and realize the necessity for such legislation, and to secure the pledge of his support of a medical bill as a condition to voting for him. We should begin at the beginning. There are something like five thousand doctors in Texas, and if organized, or if there be concert of action amongst them in this matter, they would be a power in politics.

The doctors of Ohio have taken this view of it. They have had experiences similar to ours and have tried every other plan in vain to secure medical legislation. They have at last determined upon this course, and the *Lancet Clinic* of September 28 whoops 'em up in the following language:

Don't neglect the political convention of your party. Go to it for one supreme purpose, and that is to further the legislative interests of medicine. The Ohio State Medical Society is close behind you as a strong arm. The Musgrove Bill must be enacted into a law. This is a hand-to-hand fight between the powers of evil and of righteousness, and the Ohio State Medical Society expects every man to do his whole duty.

Professional politicians and candidates for office should be given to understand that there are in Ohio five thousand regular physicians, seven hundred homeopaths and twelve hundred eclectics, who are actively arrayed in this cause. Evenly distributed throughout the State, every man a leader in his community, there are enough to endanger the political fortunes of any man who may aspire to political honors. This year the medical profession will practically act and vote as a unit. It is in their interest to do so.

